# Comprehensive analysis of the WRKY gene family in *Cucumis metuliferus* and their expression profile in response to an early stage of root knot nematode infection

**DOI:** 10.3389/fpls.2023.1143171

**Published:** 2023-03-20

**Authors:** Jian Ling, Rui Liu, Yali Hao, Yan Li, Xingxing Ping, Qihong Yang, Yuhong Yang, Xiaofei Lu, Bingyan Xie, Jianlong Zhao, Zhenchuan Mao

**Affiliations:** ^1^ Institute of Vegetables and Flowers, Chinese Academy of Agricultural Sciences, Beijing, China; ^2^ State Key Laboratory of Vegetable Biobreeding, Institute of Vegetables and Flowers, Chinese Academy of Agricultural Sciences, Beijing, China

**Keywords:** WRKY family, root knot nematode, cucumis crops, expression patterns, transcriptional regulation

## Abstract

Root-knot nematode (RKN) is a major factor that limits the growth and productivity of important *Cucumis* crops, such as cucumber and melon, which lack RKN-resistance genes in their genome. *Cucumis metuliferus* is a wild *Cucumis* species that displays a high degree of RKN-resistance. WRKY transcription factors were involved in plant response to biotic stresses. However, little is known on the function of WRKY genes in response to RKN infection in *Cucumis* crops. In this study, *Cucumis metuliferus* 60 WRKY genes (*CmWRKY*) were identified in the *C. metuliferus* genome, and their conserved domains were classified into three main groups based on multiple sequence alignment and phylogenetic analysis. Synteny analysis indicated that the WRKY genes were highly conserved in *Cucumis* crops. Transcriptome data from of *C. metuliferus* roots inoculated with RKN revealed that 16 *CmWRKY* genes showed differential expression, of which 13 genes were upregulated and three genes were downregulated, indicating that these *CmWRKY* genes are important to *C. metuliferus* response to RKN infection. Two differentially expression *CmWRKY* genes (*CmWRKY10* and *CmWRKY28*) were selected for further functional analysis. Both *CmWRKY* genes were localized in nucleus, indicating they may play roles in transcriptional regulation. This study provides a foundation for further research on the function of *CmWRKY* genes in RKN stress resistance and elucidation of the regulatory mechanism.

## 1 Introduction

In plant, WRKY genes belong to one of large plant transcription factors family that is widely involved in various physiological processes (Goyal et al., 2022). WRKY proteins contain one or two highly conserved WRKY domains that contain approximately 60 amino acid residues. The WRKY domain is located in the C-terminal region and includes a highly conserved WRKYGQK motif followed by a zinc finger motif (Kazuhiko et al., 2005). According to the number of WRKY domains and the structure of the zinc finger motif, WRKY proteins were grouped into three groups (1, 2 and 3) (Zhang and Wang, 2005). Group 1 proteins contain an N-terminal and a C-terminal WRKY domains with C2H2 motif. Group 2 proteins contain one WRKY domain but can be classified into five subgroups (2a-2e) according to C2H2 zinc-finger motif structure (Grzechowiak et al., 2022). Group 3 proteins also have one WRKY domain, but the structure of zinc-finger is C2-H-C (Ning et al., 2017). With the release of many plant genomes, whole-genome identification of WRKY genes has been reported in many plant species, and the number of WRKY family members in a species varies from dozens to hundreds (He et al., 2016; Liu et al., 2017; Karkute et al., 2018; Li et al., 2018; Chen et al., 2020; Wei et al., 2022; Zhang et al., 2022; Pan et al., 2023; Ren et al., 2023).

WRKY transcription factors are extensively involved in plant development and growth, and abiotic and biotic stresses responses by binding to the conserved motif in the promoter of their target genes (Duan et al., 2007). One focus of research on WRKY genes is their roles in regulating plant defense against biotic stress (Wani et al., 2021). WRKY genes play important roles in the plant response to diverse pathogens, including bacterial pathogens (Kim et al., 2008; Vo et al., 2018), fungal pathogens (Zheng et al., 2006) and nematodes (Chinnapandi et al., 2017). A function of WRKY genes is to regulate PAMP (pathogen-associated molecular pattern) triggered immunity (PTI) and effector-triggered immunity (ETI). WRKY transcription factors play important roles in the pathways that can activate PTI and ETI (Eulgem, 2006; Jones and Dangl, 2006; Eulgem and Somssich, 2007). In addition, WRKY transcription factors can regulate plant hormone signaling and involved in biotic stress responses. Plant WRKY genes can response to various plant hormones including ethylene, jasmonate, salicylic acid (SA), gibberellin and abscisic acid (Dai et al., 1999; Zhang et al., 2009; Grunewald et al., 2012). WRKY genes can regulate plant biotic defense mechanisms through other pathways. For example, WRKY transcription factors are responsive to regulate biosynthesis of lignin, phosphate acquisition, and other secondary metabolites (Wang et al., 2010; Schluttenhofer and Yuan, 2015; Yang et al., 2016).

Previous studies have shown that WRKY genes play important roles in plant-cyst nematode interactions. Grunewald et al. (2008) reported that knock down WRKY23 expression can reduces infection by the cyst nematode (Grunewald et al., 2008). Yang et al. (2017) reported that overexpression of three WRKY transgenes increases resistance to the cyst nematode (Yang et al., 2017). Huang et al. (2022) conducted soybean RNA-seq transcriptome sequencing of soybean for compatible and incompatible reactions to cyst nematode and observed that interactions between cyst nematode infection and WRKY genes were activated (Huang et al., 2022). An increasing number of studies have shown that WRKY genes also are involved in plant-root knot nematode (RKN) interaction. The tomato WRKY gene *SlWRKY45* is a RKN-responsive gene that enhances susceptibility to RKN (Chinnapandi et al., 2017). Tomato *SlWRKY3* can activate defense-signaling pathways associated with lipids and hormone-mediated pathway and acts as a positive regulator for resistance to *Meloidogyne javanica* (Chinnapandi et al., 2019). Ribeiro et al. (2022) reported that protease inhibitors and nucleotide-binding site leucine-rich repeat WRKY genes are essential for resistance in cowpea (Ribeiro et al., 2022).

*Cucumis* genus contains two important crops: melon and cucumber. Infections by RKN species cause heavy losses of cucumber and melon. RKN-infections seriously restricts the production and quality of cultivated cucumber and melon, due to the genome lack RKN-resistance genes (Guan et al., 2014). *Cucumis metuliferus* is considered to be a semi-wild or wild relative of melon and cucumber (Weng, 2010). Although cucumber and melon are highly susceptible to RKN infection, several *C. metuliferus* accessions are highly resistant to RKN species, including *Meloidogyne javanica* and *Meloidogyne incognita* (Walters et al., 2006; Ling et al., 2021). However, few genes involved in RKN-resistance in *C. metuliferus* have been reported. Given the lack of RKN-resistance genes in the melon and cucumber genomes, the identification of genes associated with RKN resistance in their wild relative *C. metuliferus* is of great importance. In this study, we identified 60 *CmWRKY* genes and classified them into three groups. Comparative genome analysis indicated that WRKY genes are highly conserved in *C. metuliferus*, cucumber and melon. In addition, the expression profile of *CmWRKY* genes responsive at early stage of RKN infection was investigated. The results will provide a clue for further research on the WRKY genes that regulate RKN-resistance in *Cucumis* crops.

## 2 Materials and methods

### 2.1 Genome-wide identification WRKY genes

The genome data of *C. metuliferus* were downloaded from CuGenDB (Zheng et al., 2019). HMMER program was used to search candidate WRKY genes using the WRKY domains (PF03106.7) and the e-valve was set to 1e-10 (http://hmmer.org/). The SMART software (http://smart.embl.de/) was used to prove WRKY domains in the candidate genes.

### 2.2 Gene structure, conserved motif and phylogenetic analysis

The WRKY domains of *CmWRKY* proteins and seven selected *Arabidopsis* WRKY (*AtWRKY*) proteins were used to create multiple protein sequence alignments using ClustalW2 (https://www.ebi.ac.uk/Tools/msa/clustalw2/) with default settings. The conserved motifs of CmWRKY proteins were identified using MEME software (http://meme-suite.org/) with setting –nmotifs to 10. MEGA11 (Tamura et al., 2021) was used to construct phylogenetic trees for WRKY domains. Bootstrap analysis was performed with 1000 replications to evaluate support rate.

### 2.3 Chromosomal location, gene duplication, and syntenic analysis of *CmWRKYs*

The locations of the *CmWRKYs* were extracted from the *C. metuliferus* genome gff3 annotation files and a custom Python script was used to display the distribution of *CmWRKYs* on the chromosomes. MCScanX was used to detect the duplication events of *CmWRKY*s. The syntenic analysis among *C. metuliferus*, cucumber and melon were constructed using Bioconda JCVI package (Tang et al., 2008). The WRKY genes of melon and cucumber were obtained from CuGenDB (Zheng et al., 2019).

### 2.4 Plant materials and RKN (*Meloidogyne incognita*) treatment

The RKN-resistant *C. metuliferus* were planted in greenhouse with temperature of 25°C (day) and 18°C (night). *Meloidogyne incognita* was maintained on susceptible water spinach in a glasshouse at 22–26°C. J2 juveniles of *Meloidogyne incognita* were used as inoculate. J2 juveniles were resuspended in dH2O, and the concentration of *Meloidogyne incognita* used for inoculation was 1,000/mL.

### 2.5 Transcriptome analysis, differentially expressed genes identification and expression validation by qRT-PCR

For RNA-seq, *Cm3* roots inoculated with *Meloidogyne incognita* were used as treatment and inoculated with dH_2_O were used as control. Two time points (3 day after inoculate (3DAI) and 7DAI) were selected to analysis *CmWRKYs* expression profiling at early stage of *Meloidogyne incognita* infection. *Cm3* roots were used to RNA-seq (Hiseq 2500) and performed on three independent biological replicates for each time point. The RNA-seq data processing and DEGs analysis were carried out using salmon pipeline (Patro et al., 2017) and DESeq2 (Love et al., 2014). By comparing the treatment with control at the same time point, the genes were identified as DEGs with adjusted P<0.01 and log2FoldChange >1.

We performed real-time RT-PCR (Qrt-PCR) using cfx96 real time system (Bio-Rad, USA). The PCR program was set to initial denaturation at 94°C for 3 minutes followed by 35 cycles of denaturation at 94°C for 30 s, annealing at 55 or 60°C for 30 s and extension at 72°C for 30s. The RT-PCR experiments were carried out at least three biological replicates. Besides of 3DAI and 7DAI samples, other two time points (14 DAI and 28DAI) were added in qRT-PCR experiment, which were late stage of *Mi* infection. The analysis of relative gene expression data were carried out according to Ling et al (Walters et al., 2006).

### 2.6 Subcellular localization of *CmWRKYs*

The *CmWRKY10* and *CmWRKY28* CDS sequence were cloned into the super1300-GFP vector to generate super1300- *CmWRKY10*-GFP and super1300- *CmWRKY28*-GFP. These vectors were used to transform *Agrobacterium tumefaciens* GV3101. *Nicotiana benthamiana* were grown for 4-week-old and infiltrated with transformed *A. tumefaciens* GV3101 through in infiltration buffer (10 mM MgCl_2_, 10 mM MES, and 0.1 mM acetosyringone) to an OD_600_ of 0.5. Leaves were imaged 48h after inoculation; images were acquired by an inverted confocal microscope (Zeiss LSM700, Germany) at 488 nm for GFP.

## 3 Results

### 3.1 Genome-wide identification of WRKY genes in *C. metuliferus*

**Figure 1:**
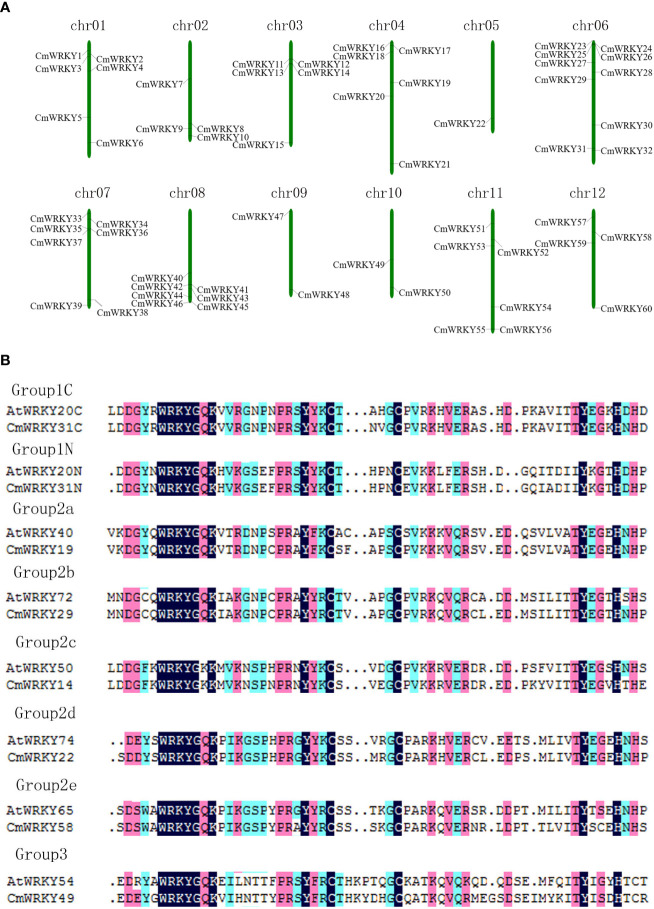
Mapping of the WRKY genes to *C*. *metuliferus* chromosomes **(A)** and alignment of amino acid sequences for selected *CmWRKY* and *AtWRKY* domain **(B)**. **(A)** to simplify the presentation, we renamed the putative WRKY genes from *CmWKRY1* to *CmWRKY60* based on their order on the chromosomes. **(B)** alignment was performed using Clustal W. The suffix ‘N’ or ‘C’ indicates the N-terminal WRKY domain or the C-terminal WRKY domain, respectively. The conserved WRKY amino acid signature is highlighted in different colors, and gaps are marked with dots.

To identify WRKY family genes in the *C. metuliferus* genome, *WRKY* genes of cucumber and melon were used to search against the *C. metuliferus* genome. The homologous proteins were identified as candidate *CmWRKY* genes using HMMER and SMART software. Finally, 60 *CmWRKY* genes in *C. metuliferus* genome were identified. These genes were predicted to contain WRKY domains based on search of the Pfam and SMART databases. All *CmWRKY* genes can be mapped to 12 chromosomes of *C. metuliferus*. Accordingly, we renamed the *CmWRKY* genes from *CmWRKY1* to *CmWRKY60* according to their location order from chromosome 1 to 12 (**Figure 1A**; **Table S1**). The distribution of *CmWRKY* genes on the chromosomes was not evenly, with chr06 containing the highest number of *CmWRKY* members (10), followed by chr07 (7) and chr08 (7), whereas chr05 contained only one *CmWRKY* gene. In order to conduct a comparative analysis among *Cucumis* genus, we mapped 60 cucumber WRKY genes (*CsWRKY*) and 58 melon WRKY genes (*MeWRKY*) to their chromosomes, respectively (**Tables S2**, **S3**, **Figures S1**, **S2**). It was noted that no WRKY gene cluster was detected in their genomes, indicating the lack of tandem replication events of WRKY genes loci in *Cucumis* species.

### 3.2 Classification and phylogenetic analysis of the *CmWRKY genes*

**Figure 2:**
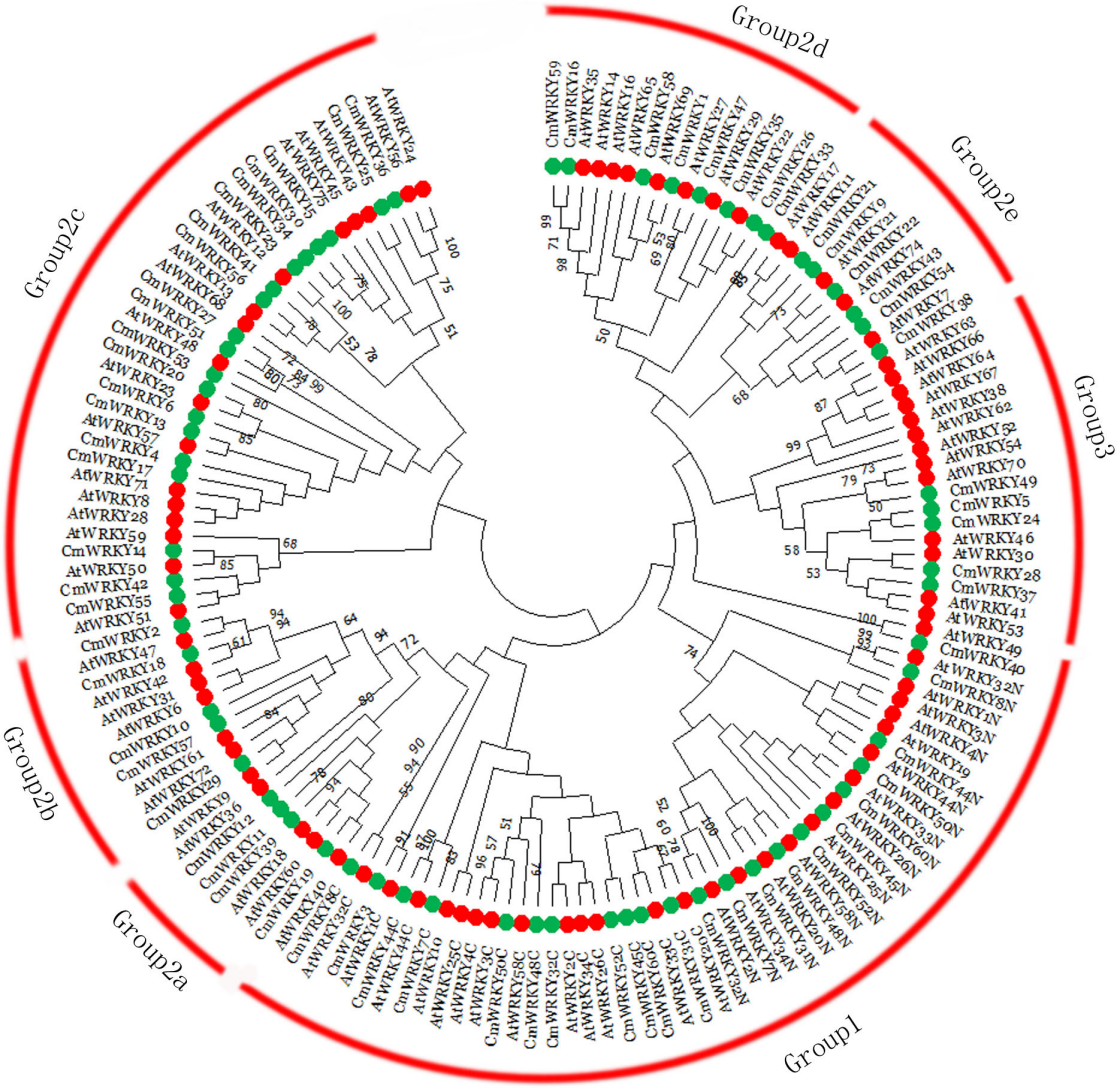
Phylogenetic tree representing relationships among WRKY domains of *Cucumis metuliferus* and *Arabidopsis thaliana*. The amino acid sequences of the WRKY domains of all *CmWRKY* and *AtWRKY* proteins were aligned with Clustal W. The phylogenetic tree was constructed using the neighbor-joining method in MEGA. The red arcs indicate different groups (or subgroups) of WRKY domains. Colored circlesrepresent putative orthologs from *C. metuliferus* (green) and Arabidopsis (red).

Multi-sequence alignment analysis of *CmWRKY* proteins was conducted using the WRKY domains. According to the group or subgroup information of *Arabidopsis* WRKY (*AtWRKY*), seven *AtWRKY* domains were randomly selected as group or subgroup representatives for the analysis. As shown in **Figure 1B** and **Figure S3**, the highly conserved amino acid sequence WRKYGQK was detected within 57 *CmWRKY* proteins, whereas the remaining three *CmWRKY* proteins (*CmWRKY14*, *CmWRKY42*, and *CmWRKY55*) had a WRKYGKK motif sequence. To understand the phylogenetic relationships of *CmWRKY* proteins, a phylogenetic tree for *CmWRKY* and *AtWRKY* WRKY domains was constructed. As shown in **Figure 2**, the *CmWRKY* genes were grouped into three large groups (Groups 1-3). Among the 60 *CmWRKY* proteins, 12 were assigned to group 1, 43 belonged to group 2, and five were assigned to group 3. The phylogenetic tree of the *CmWRKY* domains indicated that the group 2 *CmWRKY* proteins can be further grouped into five distinct subgroups (a-e). In addition, we also constructed a phylogenetic tree using WRKY gene sequences from *C. metuliferus*, cucumber (*CsWRKY*) and melon (*MeWRKY*). The number of WRKY gene family members in the three *Cucumis* crops genomes was almost identical, and the classification of the genes was similar, indicating that WRKY genes are highly conserved in these *Cucumis* crops (**Figure S4**).

### 3.3 The *CmWRKY* gene structure and conserved motifs analysis

**Figure 3:**
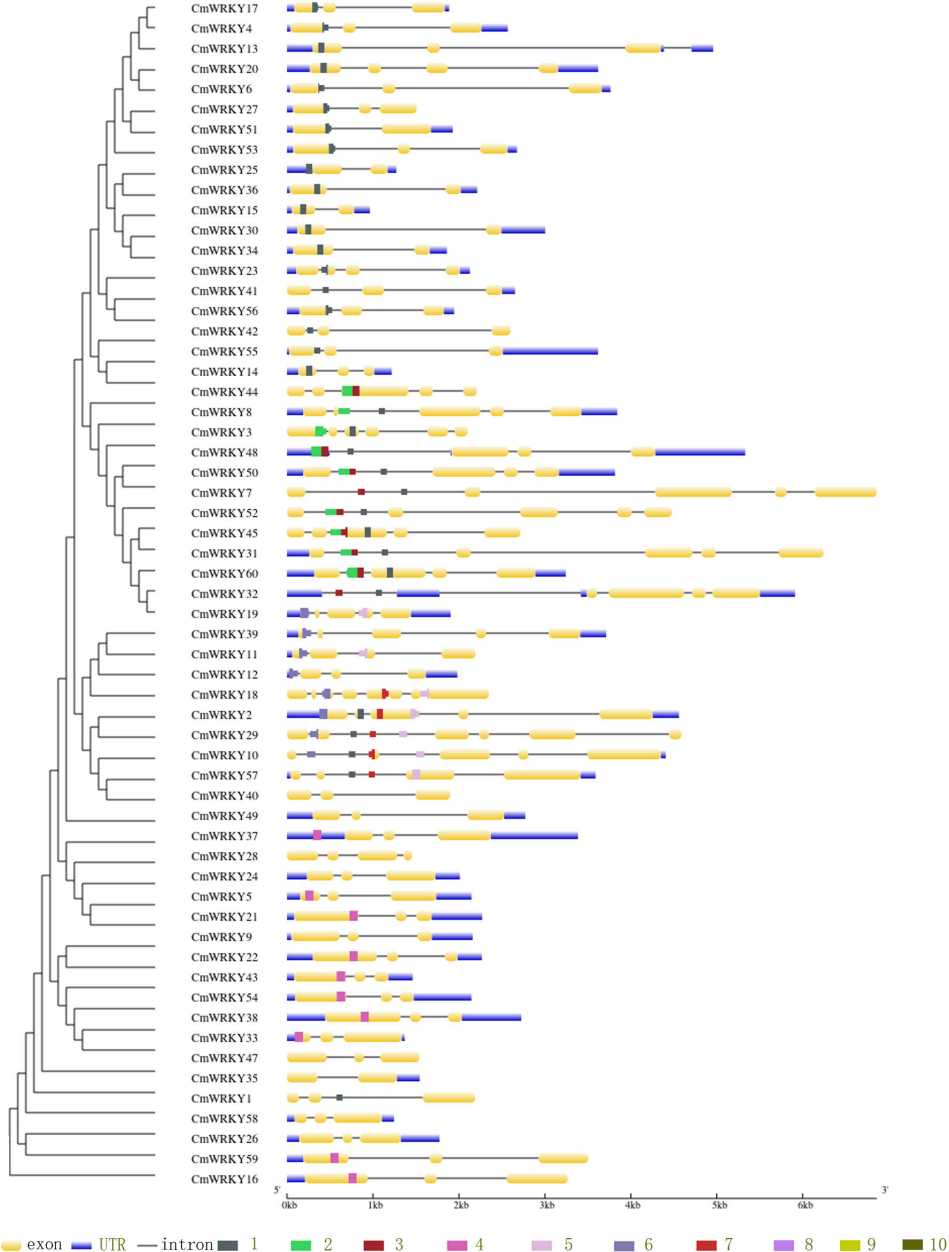
The gene structure and conserved motifs of CmWRKY genes. Left panel: the phylogenetic tree of CmWRKY domain. Right panel: the gene structure and conserved motifs. The exons, UTR and introns are indicated by yellow boxes, black lines and blue boxs, respectively.The motifs are represented by different colored boxes from number 1-10.

To detect the *CmWRKY* gene structure variations, the intron and exon structure compositions of *CmWRKY* were analyzed. As shown in **Figure 3**, among the coding sequences of *CmWRKY* genes, 27 (approximately 45%) had 3 exons, 10 *CmWRKY* genes contained 4 exons, and one WRKY gene (*CmWRKY18*) had the highest number (8) of exons. The average exon number for per *CmWRKY* gene is 3.5, while the average number of *CmWRKY* gene in some groups were significantly higher than the overall average number, with 5.6 for group 2b, 4.9 for group 1 and 4.5 for group2a members.

To evaluate the structural diversification and conservation of *CmWRKYs*, the different motifs of all *CmWRKY* proteins were identified using MEME. As shown in **Figure 3**, motif1, motif2 and motif3 were the most conserved motifs, and motif1 is shared by all *CmWRKYs*, while motif9 is the rarest which is shared by only 5 members(*CmWRKY2,10,18,29* and *57*) of group 2b (**Table S4**). Each member of *CmWRKYs* family contains 3.6 motifs on average, while group 2b members contain the largest number of motifs (6), followed by group 1 *CmWRKYs*(4.4) and group 2e (4). It is noted that, in the same phylogenetic clade, the motifs of *CmWRKY* proteins exhibited similar compositions and distributions, suggesting that *CmWRKY* genes of the same clade may be genes with similar functions.

### 3.4 Synteny analysis of *CmWRKY genes*

**Figure 4:**
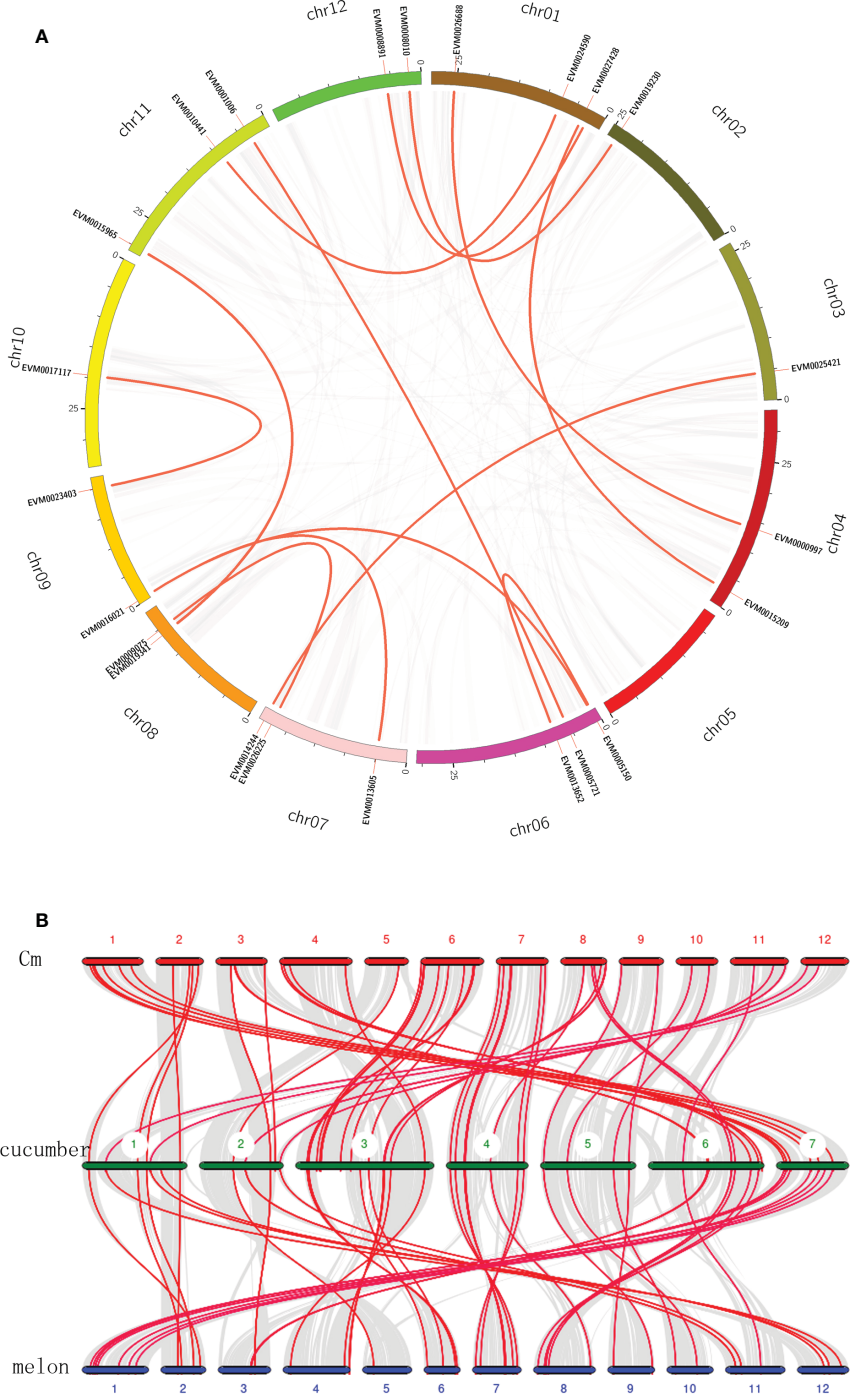
**(A)** Schematic representations of inter-chromosomal relationships of *CmWRKY* genes. Gray lines indicate synteny blocks in the *Cucumis metuliferus* genome, duplicated WRKY gene pairs are connected with red lines. **(B)** Synteny analysis of WRKY genes among *Cucumis metuliferus*, cucumber and melon. Gray lines in the background indicate the collinear blocks within these three *Cucumis* crops, and the red lines highlight the syntenic WRKY gene pairs. Cm represents *C*. *metuliferus*.

To further explore the expansion and evolution of *CmWRKY* genes, the gene duplication events within the *CmWRKY* gene family were analyzed by conducting a genome synteny analysis. Thirteen recent duplication events involving 23 *CmWRKY* genes were detected (**Figure 4A**). In contrast, tandem duplication events were not observed among *CmWRKY* genes. These results indicated that some *CmWRKY* genes were possibly derived from genome segmental duplication events and segmental duplication events play an important role in the evolution of *CmWRKY* genes.

To examine the potential evolutionary relationships among *CmWRKY* genes, *CmWRKY*, *CsWRKY* and *MeWRKY* genes were used to conduct comparative syntenic analysis. Fifty-three *CmWRKY* genes and 52 melon WRKY genes showed syntenic relationships with CsWRKY genes, respectively (**Figure 4B**). A total of 48 syntenic relationships were detected among *C. metuliferus*, cucumber and melon WRKY genes, suggesting that most WRKY genes in the three genomes were conserved. Combing syntenic and phylogenetic analyses, all WRKY genes in these three *Cucumis* genomes displayed orthologous or paralogous gene relationships. No species-specific WRKY genes were detected, indicating that no new WRKY genes had evolved among *Cucumis* crops after their divergence from *Citrullus* genus.

### 3.5 Expression patterns of *CmWRKY* genes at an early stage of RKN infection

**Figure 5:**
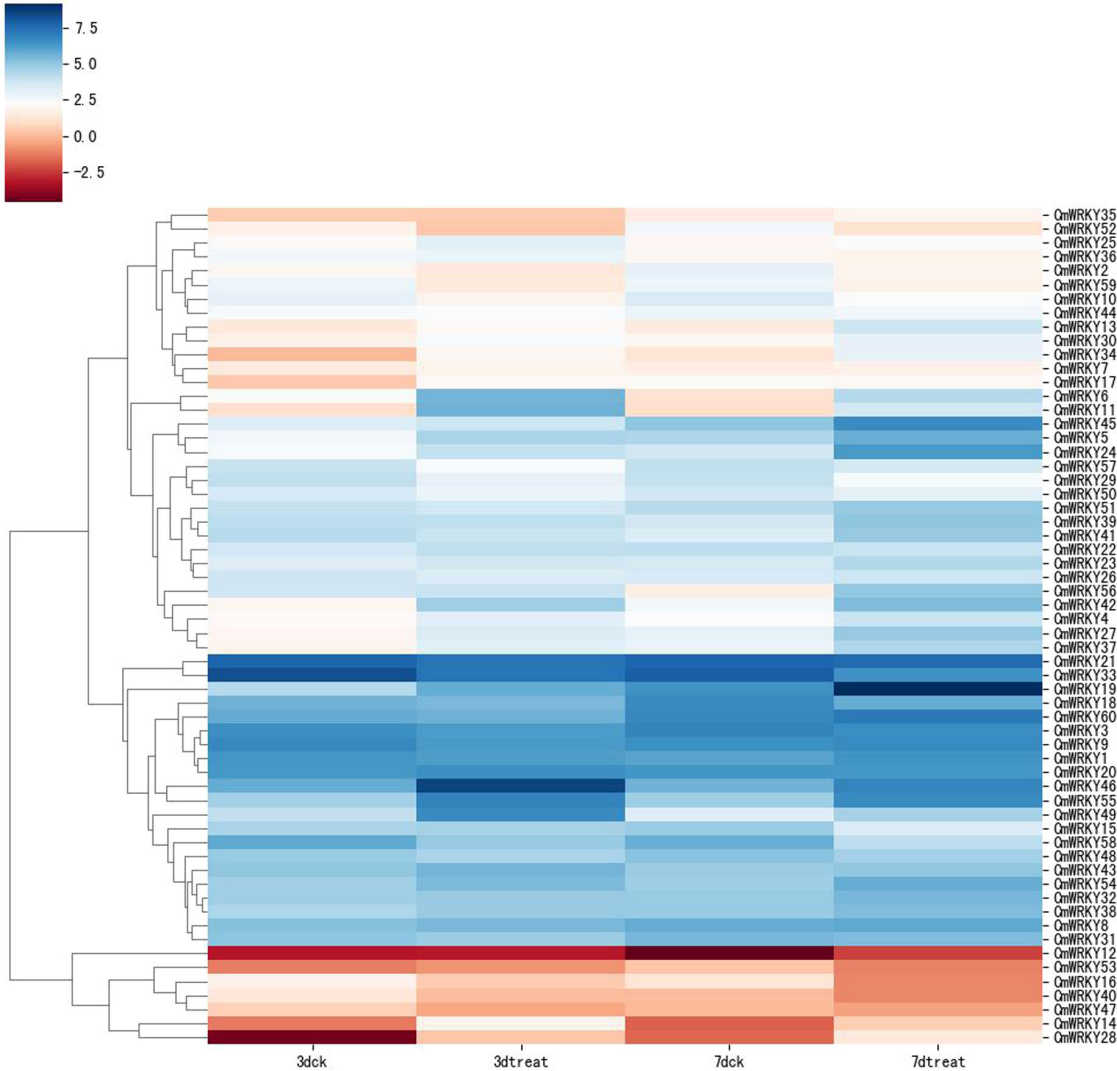
Expression pattern of *CmWRKY* genes in roots of *Cucumis metuliferus* in response to *Meloidogyne incognita* inoculation. The transcript levels of *CmWRKY* genes at 3 and 7DAI were investigated based on transcriptome data. The expression level of *CmWRKY* genes is shown as a heatmap using TPM value (log2). 3D-CK and 7D-CK represent the control of at 3DAI and 7DAI, respectively. The color scale shows increasing expression levels from red to blue.

**Figure 6:**
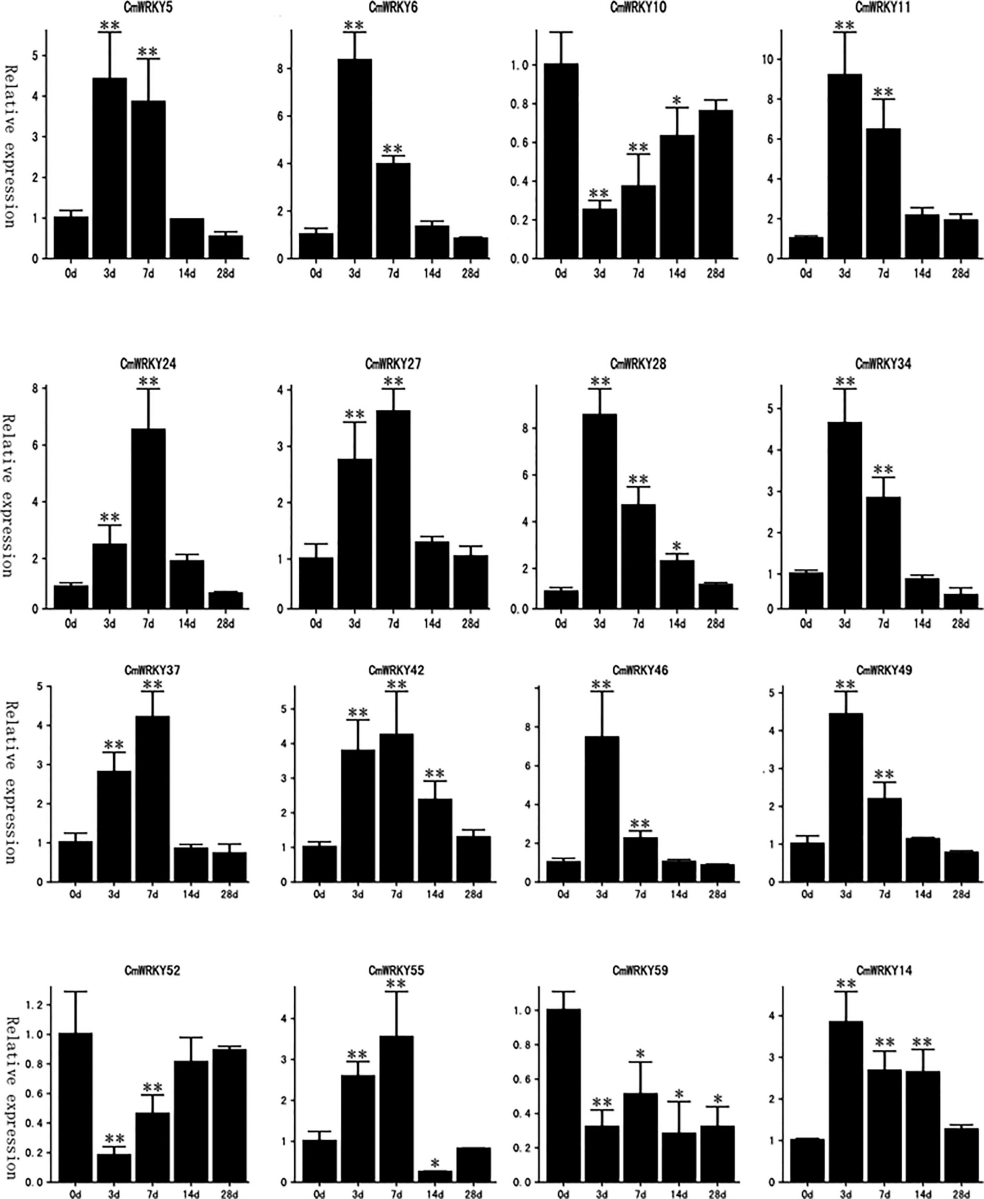
Real-time PCR detection of the expression patterns of 16 *CmWRKY* genes in roots of *Cucumis metuliferus* response to *Meloidogyne incognita* inoculation. The relative expression (y-axis) was calculated in accordance with the description in the Methods. The time points 0d, 3d, 7d, 14d, and 28d (x-axis) indicate the time(days) after inoculation. The error bars were calculated based on three replicates. Stars indicate a significant differences compared with the control (0d) (*p<0.05, **p<0.01).

Transcriptome data from *C. metuliferus* roots inoculated with *M. incognita* at an early stage of infection (3 and 7 days after infection (DAI) were used to detect the expression profile of *CmWRKY* genes in response to RKN infection. A total of 16 *CmWRKY* genes displayed a differential expression pattern at corresponding time points (3 or 7DAI), of which 13 *CmWRKY* genes were significantly upregulated, and three *CmWRKY* (*CmWRKY10, 52* and *59*) genes were significantly downregulated (p<0.01) (**Figure 5**). The expression of these differentially expressed genes (DEGs) were further verified by real-time PCR (RT-PCR). The results of RT-PCR were basically consistent with the RNA-sequencing (RNA-seq) (**Figure 6**; **Table S5**). However, in some instances, the differential expression in the RNA-seq data was significantly higher than that observed by RT-PCR. For example, at 3 DAI, the RNA-seq data showed indicated that *CmWRKY28* expression was 24 times higher compared with that of the control, whereas RT-PCR indicated that expression increased only eight times. It was noted that all DEGs did not show differential expression at 14 and 28 DAI, indicating that these WRKY DEGs only respond to RKN infection at an early stage of infection.

### 3.6 Subcellular localization of *CmWRKY* proteins

**Figure 7:**
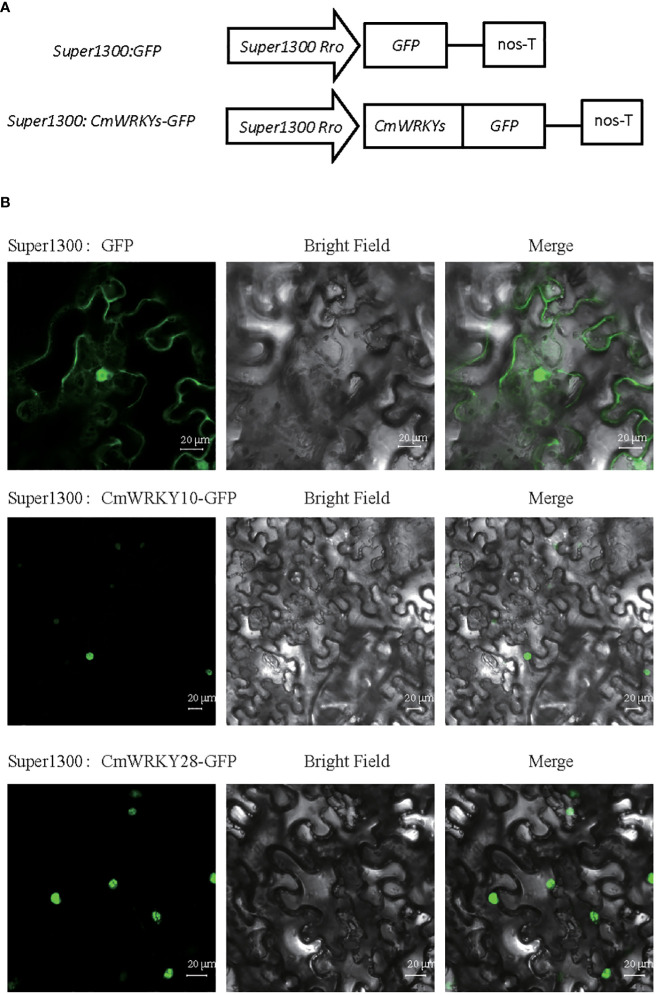
Subcellular localization analysis of *CmWRKY10* and *CmWRKY28* genes in tobacco leaf cells. **(A)** Schematic diagram of the control (super1300:GFP) and super1300:*CmWRKYs*-GFP fusion protein. **(B)** Transient expression of super1300: GFP and super1300:*CmWRKYs*-GFP in the tobacco leaf. A 48 h of after inoculation, green fluorescence signal was observed by laser confocal fluorescence microscopy.

In this study, we selected the downregulated *CmWRKY10* and the upregulated *CmWRKY28* to investigate WRKY gene function in response to RKN infection (**Figure 7A**). *CmWRKY10* and *CmWRKY28* proteins were predicted to be localized nucleus, using protein PredicProtein software. To verify prediction for two *CmWRKY* proteins, the fusion GFP proteins vectors super1300:-*CmWRKY10*-GFP and super1300:-*CmWRKY28*-GFP were transiently expressed in *N. benthamiana* leaves. The signal from the green fluorescent protein (GFP) signal was observed with a laser-scanning confocal microscopy. The epidermal cells from leaves expressing the respective *CmWRKY* fusion protein were only detected in the nucleus. In contrast, super1300: GFP was detected in the cytoplasm and the nucleus (**Figure 7B**). These data suggested that the *CmWRKY10* and *CmWRKY28* proteins were nuclear localized proteins, which was consistent with the forementioned predictions. The two *CmWRKY* transcription factors may play roles in the response of *C. metuliferus* to RKN infection.

## 4 Discussion

There are several economically important crops in the *Cucumis* genus, such as melon and cucumber. Owing to the lack of RKN-resistance genes in the cucumber and melon genomes, RKN seriously restricts the yield and quality of cultivated melon and cucumber (Weng, 2010). *C. metuliferus* is a wild relative of cucumber and melon that shows a high degree of resistance to RKN (Walters et al., 2006). The resistance of *C. metuliferus* to RKN is attained through inhibition of the development and growth of RKN at the J2 stage (Ling et al., 2021). However, the resistance mechanism of *C. metuliferus* remains unclear, and the genes involved in the interactions between *C. metuliferus* and RKN have not been identified. With publication of the *C. metuliferus* genome (Ling et al., 2017), it is feasible to identify important pathogen-resistance gene using the *C. metuliferus* genomic data. WRKY genes are important transcription factors family that are extensively involved in plant response to biological stress. In this study, we identified 60 *CmWRKY* genes in the *C. metuliferus* genomes. The number of *CmWRKY* genes is similar to that in the genomes of cucumber (60) and melon (58). It was noted that the member number of WRKY genes in *C. metuliferus*, cucumber, and melon is significantly lower than that of *Arabidopsis* (72) and rice (101) (Abdullah-Zawawi et al., 2021). No genome-wide duplication events were detected in the reported genomes of *C. metuliferus*, cucumber and melon. This can be attributed to lack of segmental duplication and tandem duplication events in the genomes of *Cucumis* crops genome (Ling et al., 2011; Chen et al., 2020). Genome duplication is a source of new genes that assume novel functions during evolution (Lawton-Rauh, 2003). Given the lack of genome duplication events, some new WRKY genes with new functions may be absent in *Cucumis* crops compared with other plants, such as *Arabidopsis* and rice. In the present study, we observed that WRKY genes were highly conserved in *C. metuliferus*, cucumber and melon. Most of the WRKY genes (48) displayed a syntenic relationship and no species-specific WRKY genes were detected in any of genomes. Taken together, these results indicated that the WRKY genes in *Cucumis* might retain the functions of the ancestral WRKY genes in plants.

Ling reported that NBS-LRR genes, which are an important pathogen-resistance genes, comprise more members (104) in *C. metuliferus* compared with those in cucumber (66) and melon (67) (Ling et al., 2017). Insertion/deletion (indel) events in the *Cucumis* genome have deleted many NBS-LRR genes in cucumber and melon. However, we did not detect indel events in the WRKY gene loci in the three genomes. Unlike NBS-LRR genes, WRKY genes not only function in disease and pathogen resistance, but also participate widely in various important physiological activities of plants. Thus, the deletion of WRKY genes may severely affect the physiology of plants. The number of WRKY gene members in *C. metuliferus*, cucumber and melon remained essentially unchanged.

Recent studies have revealed that WRKY genes are involved in plant-nematode interactions (Chinnapandi et al., 2019; Ribeiro et al., 2022). Most of this research has focused on tomato and soybean. The response of WRKY genes to nematode infection in cucurbitaceous crops has not been reported previously. A previous transcriptomic study of *C. metuliferus* roots infected by RKN has showed that plant hormone-related genes are involved in the response to RKN infection and that cytoskeleton-related genes are crucial regulators of *C. metuliferus* resistance to RKN (Ling et al., 2011). However, this study did not report on whether *CmWRKY* genes responded to nematode infection. In the present study, we analyzed the response of WRKY genes at an early stage of RKN infection, and identified 16 differentially expressed WRKY genes, of which 13 were upregulated and three were downregulated. The present expression analysis showed that *CmWRKY* genes are involved in the early response of the plant to nematode infection. We identified the orthologous genes of *CmWRKY10* and *CmWRKY28* in cucumber, which corresponds to Csav3_3g021980 and CsaV3_1G002180, respectively. The expression profiles of the two *CsWRKY* genes under RKN treatment were investigate, and no significantly differential expression can be detected for the two genes under RKN treatment in 3DAI and 7DAI (**Figure S5**). The results indicated that the differential expression of *CmWRKY* genes was related to nematode resistance. This is the first report on the response of WRKY genes to nematode in a cucurbitaceous crop. Therefore, this research enhances our understanding of WRKY gene regulation of plant resistance to RKN. However, the downstream target genes regulated by WRKY genes need to be identified to further clarify the anti-nematode function of WRKY genes.

## 5 Conclusion

Due to lack of root knot nematode (RKN) resistant genes in their genome, two important *Cucumis* crops melon and cucumber are suffered from serious RKN disease. *Cucumis metuliferus* is a wild *Cucumis* species, which displays a high level of RKN-resistance. WRKY transcription factors were involved in plant response to biotic stresses. In this study, we identified a total of 60 WRKY genes in *Cucumis metuliferus* genome. Syntonic analysis indicated that WRKY genes are highly conserved in *Cucumis metuliferus*, cucumber and melon. The expression patterns of *CmWRKY* genes under RKN stress treatment were also investigated, and 16 *CmWRKY* genes displayed differential expression at early stage of RKN infection were identified, in which two selected *CmWRKY* genes were located on nuclear by subcellular localization analysis. This is first report on WRKY genes expression profile under RKN stress in Cucumis crops. This study provided clues for further research on *CmWRKY* function in RKN-resist mechanism. Analysis of RKN-responding gene in RKN-resist Cucumis metuliferus can also provide clues to explore resistant genes in RKN-sensitive cucumber and melon.

## Data availability statement

The datasets presented in this study can be found in online repositories. The names of the repository/repositories and accession number(s) can be found below: https://ngdc.cncb.ac.cn/, PRJNA330972.

## Author contributions

JL, JZ and ZM conceived the project, XP and YH contributed to WRKY gene family analysis, RL and QY contributed to functional experiment, YY, XL and BX contributed to experiment design. All authors contributed to the article and approved the submitted version.
