# Supplementary material for: Comprehensive analysis of the WRKY gene family in Cucumis metuliferus and their expression profile in response to an early stage of root knot nematode infection
Source: Front Plant Sci. 2023 Mar 20;14:1143171. doi: 10.3389/fpls.2023.1143171 (PMC10067755; doi:10.3389/fpls.2023.1143171)
Supplement: Supplementary file 1 [file DataSheet_1.pdf]

Table S1 WRKY genes in *Cucumis metuliferus*

Geneid	*CmWRKYs*	Geneloci	groups	exons
EVM0008448	*CmWRKY1*	chr01:2551431:2553618:-	2d	3
EVM0027428	*CmWRKY2*	chr01:3394987:3399547:-	2b	5
EVM0010330	*CmWRKY3*	chr01:3932880:3934980:+	1	6
EVM0024590	*CmWRKY4*	chr01:7645412:7647979:-	2c	3
EVM0007950	*CmWRKY5*	chr01:19191637:19193782:+	3	3
EVM0026688	*CmWRKY6*	chr01:25442476:25446239:+	2c	3
EVM0020788	*CmWRKY7*	chr02:9554781:9561641:+	1	5
EVM0002012	*CmWRKY8*	chr02:20743754:20747594:+	1	5
EVM0013493	*CmWRKY9*	chr02:21964020:21966179:-	2e	3
EVM0019230	*CmWRKY10*	chr02:23811062:23815467:-	2b	5
EVM0025421	*CmWRKY11*	chr03:4622414:4624605:+	2a	4
EVM0001958	*CmWRKY12*	chr03:4632273:4634254:+	2a	4
EVM0017429	*CmWRKY13*	chr03:5675337:5680294:+	2c	4
EVM0007128	*CmWRKY14*	chr03:5915790:5917007:-	2c	3
EVM0010564	*CmWRKY15*	chr03:25570698:25571662:-	2c	2
EVM0025595	*CmWRKY16*	chr04:501878:505142:+	2d	3
EVM0009239	*CmWRKY17*	chr04:1288492:1290378:-	2c	3
EVM0015209	*CmWRKY18*	chr04:2867760:2870108:-	2b	8
EVM0006442	*CmWRKY19*	chr04:10513779:10515683:+	2a	5
EVM0000997	*CmWRKY20*	chr04:13825280:13828900:+	2c	4
EVM0026425	*CmWRKY21*	chr04:30705278:30707548:-	2e	3
EVM0022287	*CmWRKY22*	chr05:19366072:19368337:-	2e	3
EVM0018986	*CmWRKY23*	chr06:92854:94982:+	2c	4
EVM0005150	*CmWRKY24*	chr06:777220:779231:+	3	3
EVM0019016	*CmWRKY25*	chr06:1023400:1024672:+	2c	2
EVM0003666	*CmWRKY26*	chr06:1033489:1035262:-	2d	3
EVM0005721	*CmWRKY27*	chr06:5530619:5532127:+	2c	3
EVM0013652	*CmWRKY28*	chr06:7867885:7869340:-	3	4
EVM0018335	*CmWRKY29*	chr06:9681623:9686211:+	2b	6
EVM0023472	*CmWRKY30*	chr06:21056816:21059819:-	2c	2
EVM0023050	*CmWRKY31*	chr06:26912971:26922619:+	1	6
EVM0007126	*CmWRKY32*	chr06:27413285:27419195:+	1	6
EVM0027767	*CmWRKY33*	chr07:1071323:1072691:+	2d	3
EVM0006756	*CmWRKY34*	chr07:2507183:2509042:+	2c	2
EVM0013605	*CmWRKY35*	chr07:4917306:4918849:+	2d	2
EVM0025633	*CmWRKY36*	chr07:4923436:4925648:+	2c	2
EVM0006672	*CmWRKY37*	chr07:5271343:5275641:-	3	4
EVM0026225	*CmWRKY38*	chr07:22663317:22666042:-	2e	3
EVM0014244	*CmWRKY39*	chr07:23980603:23984315:-	2a	5
EVM0025345	*CmWRKY40*	chr08:15983592:15985490:+	1	3

EVM0019341	*CmWRKY41*	chr08:18852622:18855273:-	2c	3
EVM0027240	*CmWRKY42*	chr08:18934381:18936982:+	2c	3
EVM0009075	*CmWRKY43*	chr08:19798141:19799602:-	2e	3
EVM0019152	*CmWRKY44*	chr08:21933169:21935377:+	1	5
EVM0004707	*CmWRKY45*	chr08:23134295:23137009:+	1	5
EVM0017946	*CmWRKY46*	chr08:23311594:23312546:-	2c	3
EVM0016021	*CmWRKY47*	chr09:598571:600110:-	2d	3
EVM0023403	*CmWRKY48*	chr09:20047582:20052912:-	1	4
EVM0018315	*CmWRKY49*	chr10:12637319:12640090:+	3	3
EVM0017117	*CmWRKY50*	chr10:19580561:19584376:-	1	4
EVM0001006	*CmWRKY51*	chr11:3641580:3643508:-	2c	2
EVM0024264	*CmWRKY52*	chr11:7682367:7686842:-	1	5
EVM0010441	*CmWRKY53*	chr11:9278855:9281529:-	2c	3
EVM0021173	*CmWRKY54*	chr11:24434599:24436744:+	2e	3
EVM0002825	*CmWRKY55*	chr11:29972652:29976269:-	2c	3
EVM0015965	*CmWRKY56*	chr11:30017170:30019820:+	2c	3
EVM0008010	*CmWRKY57*	chr12:2053542:2057131:-	2b	4
EVM0008891	*CmWRKY58*	chr12:5759531:5760775:+	2d	3
EVM0007057	*CmWRKY59*	chr12:8511187:8514691:-	2d	3
EVM0024169	*CmWRKY60*	chr12:24685357:24688599:+	1	5

Table S2 WRKY genes in melon

Geneid	*CmWRKYs*	Geneloci
MELO3C018717	*MeWRKY1*	chr01:2329439:2332110:-
MELO3C018826	*MeWRKY2*	chr01:3164464:3170498:-
MELO3C024209	*MeWRKY3*	chr01:4233323:4236817:+
MELO3C024135	*MeWRKY4*	chr01:5602440:5604478:-
MELO3C012635	*MeWRKY5*	chr01:21810476:21813203:-
MELO3C015776	*MeWRKY6*	chr01:29769740:29772599:-
MELO3C015910	*MeWRKY7*	chr01:31687979:31691074:-
MELO3C010057	*MeWRKY8*	chr02:12767901:12786128:+
MELO3C010223	*MeWRKY9*	chr02:15607473:15611233:+
MELO3C017415	*MeWRKY10*	chr02:23453889:23455490:-
MELO3C017157	*MeWRKY11*	chr02:25559550:25563329:-
MELO3C008175	*MeWRKY12*	chr03:1547423:1550563:+
MELO3C011432	*MeWRKY13*	chr03:26047129:26051259:-
MELO3C011296	*MeWRKY14*	chr03:27110508:27112112:-
MELO3C011295	*MeWRKY15*	chr03:27118894:27120900:-
MELO3C003622	*MeWRKY16*	chr04:2790915:2793594:+
MELO3C012932	*MeWRKY17*	chr04:14696271:14699542:-
MELO3C026740	*MeWRKY18*	chr04:25328205:25330363:-
MELO3C009302	*MeWRKY19*	chr04:32999500:33002049:+

MELO3C009127	*MeWRKY20*	chr04:34052833:34055772:+
MELO3C009097	*MeWRKY21*	chr04:34224729:34226937:-
MELO3C014305	*MeWRKY22*	chr05:4037594:4039709:+
MELO3C005937	*MeWRKY23*	chr06:85973:87936:+
MELO3C006037	*MeWRKY24*	chr06:709798:711903:+
MELO3C006078	*MeWRKY25*	chr06:911832:912675:+
MELO3C006081	*MeWRKY26*	chr06:918990:921033:-
MELO3C006725	*MeWRKY27*	chr06:5400020:5401243:+
MELO3C026932	*MeWRKY28*	chr06:8899218:8900924:+
MELO3C019494	*MeWRKY29*	chr06:11742100:11745798:+
MELO3C014896	*MeWRKY30*	chr06:21668053:21670897:+
MELO3C014066	*MeWRKY31*	chr06:36458743:36463268:-
MELO3C014134	*MeWRKY32*	chr06:37238836:37247788:-
MELO3C016966	*MeWRKY33*	chr07:797629:801138:+
MELO3C016281	*MeWRKY34*	chr07:23102770:23105302:-
MELO3C016337	*MeWRKY35*	chr07:23713196:23715444:-
MELO3C016338	*MeWRKY36*	chr07:23722485:23724246:-
MELO3C017743	*MeWRKY37*	chr07:26732547:26734045:-
MELO3C017912	*MeWRKY38*	chr07:28034739:28036194:-
MELO3C007048	*MeWRKY39*	chr08:463779:467277:+
MELO3C007157	*MeWRKY40*	chr08:1168678:1170864:-
MELO3C007409	*MeWRKY41*	chr08:2598395:2601444:-
MELO3C007470	*MeWRKY42*	chr08:3016147:3017915:+
MELO3C007576	*MeWRKY43*	chr08:3811244:3816260:-
MELO3C003140	*MeWRKY44*	chr08:32467441:32469570:-
MELO3C022014	*MeWRKY45*	chr09:1829315:1834205:+
MELO3C002875	*MeWRKY46*	chr09:6787189:6790868:+
MELO3C005843	*MeWRKY47*	chr09:24710923:24712335:+
MELO3C012130	*MeWRKY48*	chr10:2464074:2467816:+
MELO3C020162	*MeWRKY49*	chr10:13947920:13953702:+
MELO3C020166	*MeWRKY50*	chr10:13974104:13976892:-
MELO3C020967	*MeWRKY51*	chr11:2422711:2425241:-
MELO3C020963	*MeWRKY52*	chr11:2478322:2481294:+
MELO3C024787	*MeWRKY53*	chr11:8413509:8415270:-
MELO3C019679	*MeWRKY54*	chr11:24451781:24454215:+
MELO3C025686	*MeWRKY55*	chr11:27244015:27248661:+
MELO3C020489	*MeWRKY56*	chr12:210826:213856:-
MELO3C002675	*MeWRKY57*	chr12:22146043:22147343:-
MELO3C002202	*MeWRKY58*	chr12:25640685:25644463:+

Table S3 WRKY genes in cucumber

Geneid	*CmWRKYs*	Geneloci
CsaV3_1G002180	*CsWRKY1*	chr1:1407016:1410875:+
CsaV3_1G004720	*CsWRKY2*	chr1:2993098:2996444:+
CsaV3_1G007870	*CsWRKY3*	chr1:4979523:4983223:-
CsaV3_1G028960	*CsWRKY4*	chr1:15852778:15856825:+
CsaV3_1G032000	*CsWRKY5*	chr1:19017073:19019018:-
CsaV3_1G033110	*CsWRKY6*	chr1:20150144:20151996:-
CsaV3_1G037680	*CsWRKY7*	chr1:23633542:23637299:+
CsaV3_1G044520	*CsWRKY8*	chr1:29902748:29918342:+
CsaV3_2G013650	*CsWRKY9*	chr2:11212968:11215966:-
CsaV3_2G017720	*CsWRKY10*	chr2:14723306:14726183:-
CsaV3_2G017760	*CsWRKY11*	chr2:14764150:14767009:+
CsaV3_2G032460	*CsWRKY12*	chr2:21438787:21441053:+
CsaV3_2G032470	*CsWRKY13*	chr2:21447808:21449327:+
CsaV3_2G034030	*CsWRKY14*	chr2:22545696:22550853:+
CsaV3_2G035630	*CsWRKY15*	chr2:23904722:23906085:+
CsaV3_3G003840	*CsWRKY16*	chr3:3226389:3233715:-
CsaV3_3G004410	*CsWRKY17*	chr3:3723776:3732326:-
CsaV3_3G007160	*CsWRKY18*	chr3:6430612:6433611:+
CsaV3_3G008170	*CsWRKY19*	chr3:7021640:7024904:+
CsaV3_3G008580	*CsWRKY20*	chr3:7219640:7220399:+
CsaV3_3G008610	*CsWRKY21*	chr3:7225695:7227936:-
CsaV3_3G015290	*CsWRKY22*	chr3:11362956:11365199:+
CsaV3_3G018790	*CsWRKY23*	chr3:14489815:14493411:-
CsaV3_3G021980	*CsWRKY24*	chr3:19080570:19082350:-
CsaV3_3G026600	*CsWRKY25*	chr3:22733862:22737626:-
CsaV3_3G026920	*CsWRKY26*	chr3:23279533:23282478:+
CsaV3_3G033000	*CsWRKY27*	chr3:28527402:28529709:+
CsaV3_3G033350	*CsWRKY28*	chr3:28698684:28702697:-
CsaV3_3G035430	*CsWRKY29*	chr3:29704664:29706882:-
CsaV3_3G047140	*CsWRKY30*	chr3:38433423:38436416:-
CsaV3_4G001260	*CsWRKY31*	chr4:722761:724505:+
CsaV3_4G003030	*CsWRKY32*	chr4:1881152:1882944:+
CsaV3_4G006110	*CsWRKY33*	chr4:4040395:4042324:+
CsaV3_4G006120	*CsWRKY34*	chr4:4045602:4047366:+
CsaV3_4G006480	*CsWRKY35*	chr4:4328507:4332383:-
CsaV3_4G025110	*CsWRKY36*	chr4:14752304:14754434:-
CsaV3_4G034570	*CsWRKY37*	chr4:24478962:24480541:+
CsaV3_4G036610	*CsWRKY38*	chr4:25729944:25733619:-
CsaV3_5G001010	*CsWRKY39*	chr5:504127:505921:-
CsaV3_5G011080	*CsWRKY40*	chr5:7047174:7050833:-
CsaV3_5G033090	*CsWRKY41*	chr5:26596980:26600654:-
CsaV3_5G038330	*CsWRKY42*	chr5:30396040:30400995:-

CsaV3_6G013820	*CsWRKY43*	chr6:10004976:10007709:-
CsaV3_6G018960	*CsWRKY44*	chr6:13759828:13764587:-
CsaV3_6G028510	*CsWRKY45*	chr6:16783402:16791738:-
CsaV3_6G032100	*CsWRKY46*	chr6:18178380:18181433:+
CsaV3_6G032480	*CsWRKY47*	chr6:18415484:18434126:-
CsaV3_6G042200	*CsWRKY48*	chr6:24728797:24731520:-
CsaV3_6G042280	*CsWRKY49*	chr6:24809801:24812529:+
CsaV3_6G043450	*CsWRKY50*	chr6:25618485:25621280:-
CsaV3_6G048830	*CsWRKY51*	chr6:28610615:28613553:+
CsaV3_6G051490	*CsWRKY52*	chr6:29962063:29964115:+
CsaV3_6G052610	*CsWRKY53*	chr6:30645979:30650216:-
CsaV3_7G002670	*CsWRKY54*	chr7:2056294:2058276:-
CsaV3_7G003470	*CsWRKY55*	chr7:2571163:2574863:+
CsaV3_7G005750	*CsWRKY56*	chr7:3456072:3459066:+
CsaV3_7G022650	*CsWRKY57*	chr7:11502732:11505428:+
CsaV3_7G025370	*CsWRKY58*	chr7:14754543:14756982:+
CsaV3_7G027490	*CsWRKY59*	chr7:17076064:17079015:+
CsaV3_7G030110	*CsWRKY60*	chr7:19053279:19056859:+

Table S4 the motifs of *CmWRKY* genes

Genes	motifnumber	motifs
CmWRKY1	3	motif1,motif2,motif3,
CmWRKY2	6	motif3,motif9,motif2,motif7,motif8,motif1,
CmWRKY3	4	motif3,motif2,motif1,motif4,
CmWRKY4	3	motif3,motif2,motif1,
CmWRKY5	4	motif2,motif10,motif1,motif6,
CmWRKY6	3	motif1,motif3,motif2,
CmWRKY7	4	motif2,motif3,motif1,motif5,
CmWRKY8	4	motif3,motif2,motif1,motif4,
CmWRKY9	4	motif2,motif10,motif1,motif6,
CmWRKY10	6	motif8,motif7,motif1,motif2,motif9,motif3,
CmWRKY11	4	motif2,motif1,motif8,motif7,
CmWRKY12	3	motif1,motif8,motif2,
CmWRKY13	3	motif1,motif3,motif2,
CmWRKY14	3	motif1,motif3,motif2,
CmWRKY15	3	motif1,motif2,motif3,
CmWRKY16	3	motif2,motif6,motif1,
CmWRKY17	3	motif1,motif3,motif2,
CmWRKY18	5	motif7,motif8,motif1,motif9,motif2,
CmWRKY19	4	motif2,motif1,motif8,motif7,
CmWRKY20	3	motif1,motif3,motif2,

CmWRKY21	4	motif10,motif2,motif1,motif6,
CmWRKY22	4	motif2,motif10,motif6,motif1,
CmWRKY23	3	motif2,motif3,motif1,
CmWRKY24	3	motif2,motif10,motif1,
CmWRKY25	3	motif1,motif2,motif3,
CmWRKY26	2	motif1,motif2,
CmWRKY27	3	motif1,motif3,motif2,
CmWRKY28	3	motif10,motif2,motif1,
CmWRKY29	6	motif2,motif3,motif9,motif7,motif8,motif1,
CmWRKY30	3	motif2,motif3,motif1,
CmWRKY31	5	motif5,motif1,motif4,motif3,motif2,
CmWRKY32	5	motif1,motif4,motif5,motif3,motif2,
CmWRKY33	3	motif6,motif1,motif2,
CmWRKY34	3	motif3,motif2,motif1,
CmWRKY35	2	motif1,motif2,
CmWRKY36	3	motif3,motif2,motif1,
CmWRKY37	4	motif10,motif2,motif1,motif6,
CmWRKY38	4	motif2,motif10,motif1,motif6,
CmWRKY39	4	motif2,motif8,motif7,motif1,
CmWRKY40	2	motif2,motif1,
CmWRKY41	3	motif1,motif2,motif3,
CmWRKY42	3	motif1,motif2,motif3,
CmWRKY43	4	motif10,motif2,motif1,motif6,
CmWRKY44	4	motif1,motif4,motif5,motif2,
CmWRKY45	5	motif5,motif4,motif1,motif2,motif3,
CmWRKY46	2	motif10,motif1,
CmWRKY47	2	motif2,motif1,
CmWRKY48	5	motif3,motif2,motif1,motif4,motif5,
CmWRKY49	2	motif10,motif1,
CmWRKY50	5	motif2,motif3,motif5,motif4,motif1,
CmWRKY51	3	motif2,motif3,motif1,
CmWRKY52	5	motif5,motif1,motif4,motif3,motif2,
CmWRKY53	3	motif2,motif3,motif1,
CmWRKY54	4	motif2,motif10,motif6,motif1,
CmWRKY55	3	motif1,motif3,motif2,
CmWRKY56	3	motif1,motif3,motif2,
CmWRKY57	6	motif8,motif1,motif7,motif3,motif9,motif2,
CmWRKY58	2	motif2,motif1,
CmWRKY59	3	motif2,motif1,motif6,
CmWRKY60	5	motif2,motif3,motif4,motif1,motif5,

Table S5 The relative expression values of differential expression genes

	0d	0d_error	3d	3d_erro	7d	7d_error	14d	14d_error	28d	28d_error
CmWRKY5	1	0.19	4.41	1.168	3.85	1.07	0.95	0.02	0.53	0.13
CmWRKY6	1	0.28	8.34	1.19	3.95	0.38	1.32	0.26	0.82	0.085
CmWRKY10	1	0.17	0.25	0.05	0.37	0.17	0.63	0.15	0.76	0.06
CmWRKY11	1	0.14	9.18	2.18	6.44	1.56	2.14	0.41	1.89	0.34
CmWRKY24	1	0.15	2.47	0.71	6.52	1.46	1.89	0.26	0.76	0.08
CmWRKY27	1	0.27	2.75	0.68	3.61	0.41	1.28	0.12	1.04	0.19
CmWRKY28	1	0.19	8.54	1.14	4.68	0.81	2.29	0.34	1.28	0.12
CmWRKY34	1	0.09	4.64	0.84	2.83	0.51	0.86	0.11	0.48	0.19
CmWRKY37	1	0.25	2.8	0.51	4.2	0.67	0.84	0.12	0.72	0.25
CmWRKY42	1	0.16	3.78	0.91	4.24	1.27	2.36	0.56	1.28	0.23
CmWRKY46	1	0.23	7.44	2.4	2.23	0.41	1.02	0.14	0.84	0.09
CmWRKY49	1	0.22	4.42	0.62	2.18	0.46	1.12	0.06	0.76	0.07
CmWRKY52	1	0.29	-2.52	0.56	-1.42	0.13	-1.26	0.17	0.89	0.03
CmWRKY55	1	0.24	2.58	0.37	3.54	1.12	0.24	0.034	0.81	0.03
CmWRKY59	1	0.11	0.32	0.1	0.51	0.19	0.28	0.19	0.32	0.12
CmWRKY14	1	0.05	3.83	0.75	2.67	0.48	2.63	0.56	1.26	0.12
