# Supplementary material for: Comprehensive analysis of the WRKY gene family in Cucumis metuliferus and their expression profile in response to an early stage of root knot nematode infection
Source: Front Plant Sci. 2023 Mar 20;14:1143171. doi: 10.3389/fpls.2023.1143171 (PMC10067755; doi:10.3389/fpls.2023.1143171)

Figure S1 Mapping of the WRKY gene family on cucumber chromosomes

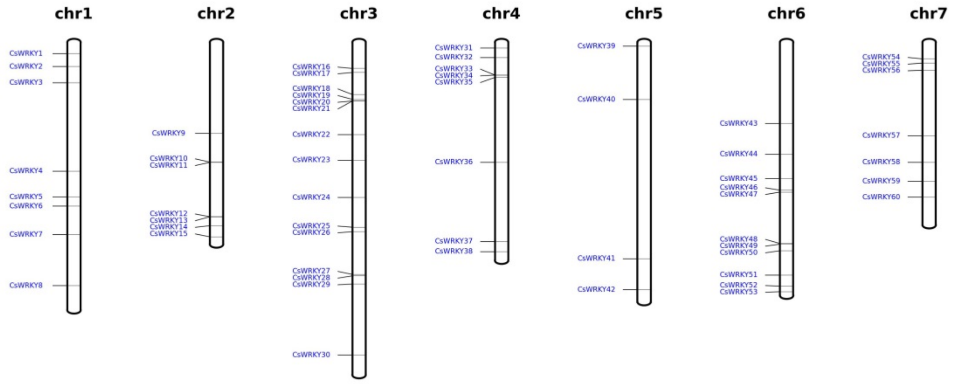

Figure S2 Mapping of the WRKY gene family on melon chromosomes

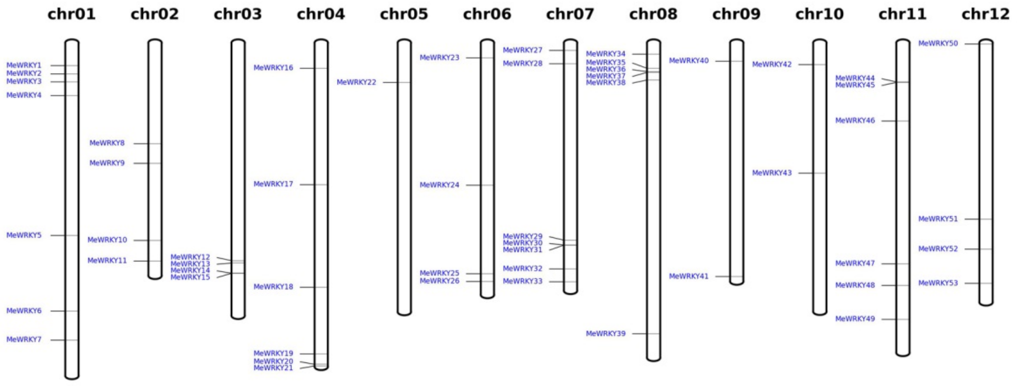

Figure S3 Alignment of multiple CmWRKY and selected AtWRKY domain amino acid sequences. Alignment was performed using Clustal W. The suffix 'N' or 'C' indicates the N-terminal WRKY domain or the C-terminal WRKY domain, respectively. The conserved WRKY amino acid signature is highlighted in colors, and gaps are marked with dots

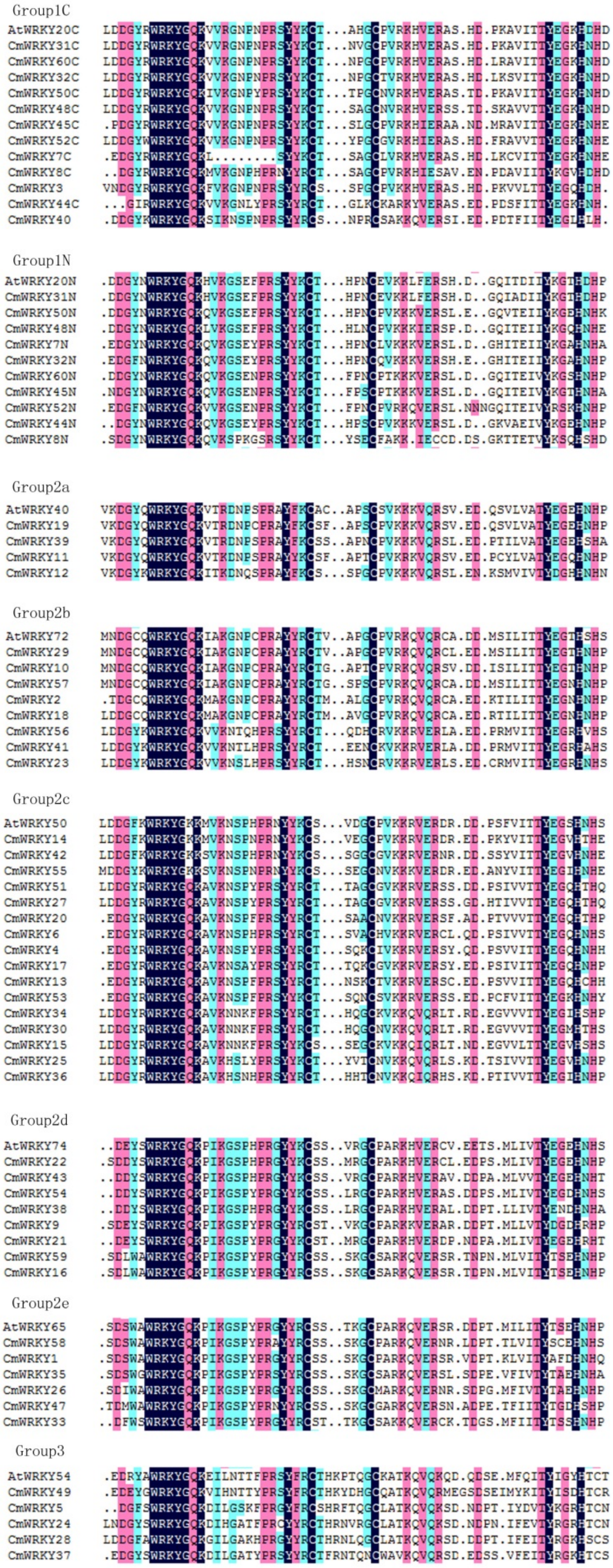

Figure S4 Phylogenetic tree representing relationships among WRKY domains of C. metuliferus , cucumber and melon. Selected Arabidopsis WRKY genes were used as representatives for analysis.

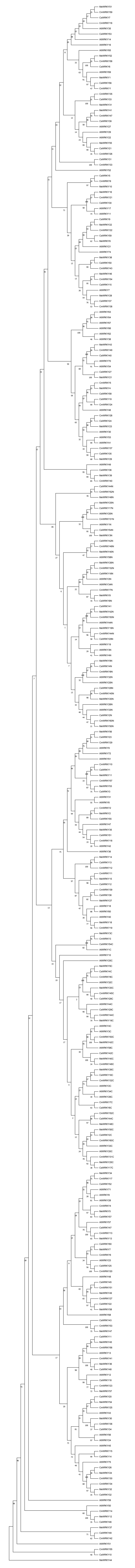

Figure S5 The exrpression analysis of two cucumber WRKY genes after RKN infection

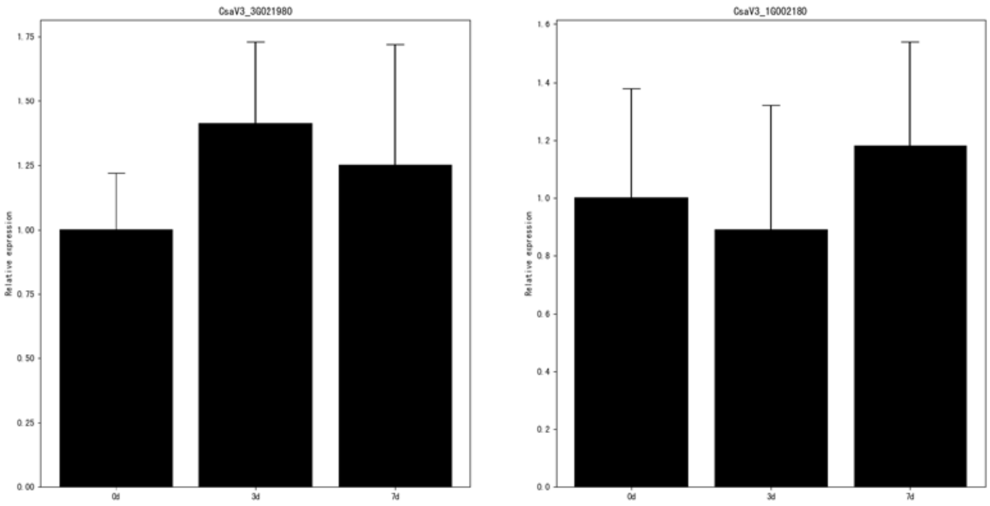

Supplement: Supplementary file 2 [file DataSheet_2.pdf]
